# Supplementary material for: Velocity-Selective Arterial Spin Labeling Perfusion in Monitoring High Grade Gliomas Following Therapy: Clinical Feasibility at 1.5T and Comparison with Dynamic Susceptibility Contrast Perfusion
Source: Brain Sci. 2024 Jan 25;14(2):126. doi: 10.3390/brainsci14020126 (PMC10886779; doi:10.3390/brainsci14020126)
Supplement: Supplementary file 1 [file brainsci-14-00126-s001.zip › brainsci-2827381-supplementary.pdf]

Table S1. Clinical Profile

pt	age	sex	group	primary tumor diagnosis (WHO grade)	dd	treatment history
**1**	63	M	RT	Glioblastoma (IV), IDH-wt, unknown MGMT	301	standard*
**2**	54	M	RT	Glioblastoma (IV), IDH-wt, MGMT methylated	2472	standard
**3**	77	M	RT	Glioblastoma (IV), IDH-wt, MGMT methylated	755	standard
**4**	51	F	RT, H+	Glioblastoma (IV), IDH-wt, unknown MGMT	416	standard + 2 surgeries + study**
**5**	56	F	RT	Anaplastic Astrocytoma (III), IDH-wt, no MGMT methylation	263	standard
**6**	22	F	RT	Anaplastic Astrocytoma (III), IDH-wt, MGMT methylated	495	standard
**7**	70	M	RT, H+	Glioblastoma (IV), IDH-wt, unknown MGMT	425	standard
**8**	28	F	RT, H+	Glioblastoma (IV), IDH-wt, unknown MGMT	2333	standard
**9**	51	M	RT	Glioblastoma (IV), IDH-wt, MGMT methylated	593	standard + surgery + study[†]
**10**	67	M	NRT	Anaplastic Astrocytoma (III), unknown IDH/MGMT	8664	radiation + PCV
**11**	43	M	NRT	Anaplastic Astrocytoma (III), unknown IDH/MGMT	3629	standard
**12**	43	M	NRT	Anaplastic Astrocytoma (III), IDH-m, unknown MGMT	2580	resection + radiation + PCV
**13**	62	M	NRT	Glioblastoma (IV), IDH-wt, unknown MGMT	225	standard
**14**	68	M	NRT	Glioblastoma (IV), IDH-wt, unknown MGMT	548	standard + surgery
**15**	79	F	NRT	Glioblastoma (IV), IDH-wt, no MGMT methylation	1535	standard

**16**	33	F	NRT	Anaplastic Infiltrating Glioma (III), IDH-m, unknown MGMT	1973	standard + Mebendazol
**17**	41	M	NRT	Anaplastic Astrocytoma (III), IDH-m, no MGMT methylation	2082	standard
**18**	66	M	NRT	Glioblastoma (IV), IDH-wt, no MGMT methylation	281	standard
**19**	68	F	NRT	Epitheloid Glioblastoma (IV), IDH-wt, MGMT methylated	121	standard
**20**	61	F	NRT	Anaplastic Astrocytoma (III), IDH-m, unknown MGMT	4141	standard
**21**	62	F	NRT	Glioblastoma (IV), IDH-wt, MGMT methylated	458	standard + surgery
**22**	46	M	NRT	Anaplastic Astrocytoma (III), unknown IDH/MGMT	4383	standard
**23**	64	F	NRT	Diffuse Oligodendroglioma (III), unknown IDH/MGMT	7165	standard
**24**	69	M	NRT	Glioblastoma (IV), IDH-wt, unknown MGMT	2364	standard + 2 surgeries
**25**	47	M	NRT	Glioblastoma (IV), IDH-wt, MGMT methylated	1878	standard + 3 surgeries + study[§]+ chemoradiation
**26**	72	F	NRT	Glioblastoma (IV), IDH-wt, unknown MGMT	1964	Standard
**27**	43	F	NRT	Anaplastic Astrocytoma (III), unknown IDH/MGMT	4318	standard + Bevacizumab
**28**	39	M	NRT	Anaplastic Astrocytoma (III), unknown IDH/MGMT	4831	standard + surgery

Pt - patient, dd - days since diagnosis, WHO - World Health Organization, M - male, F - female, RT - residual/recurrent tumor, NRT - no residual/recurrent tumor, H+ - active tumor in histopathology (within 4 weeks of imaging), IDH - isocitrate dehydrogenase, wt - wild type, m - mutated, MGMT - O-6-methylguanine-DNA methyltransferase

PCV - Procarbazine, 1-(2-chloroethyl)-3-cyclohexyl-1-nitrosourea (CCNU, Lomustine), Vincristine

* standard - 6 weeks of chemoradiation with Temozolomide (Temodar) followed by 6 cycles of adjuvant Temozolomide

** N1604 - Phase 0/I study of AMG 232 at 240 mg 1x daily for 7 consecutive days every 21 days

† J1444 - Glioma Atkins-based Diet study

§ J11120 - Phase II trial with chemo-sensitizing low-dose radiation for 6 months (Radtx 0.5 Gy twice daily on Days 1-5 of the normal 28-day Temodar cycle)
